# One-step enzymatic synthesis of medium molecular weight dextran using engineered dextransucrase DarM from *Leuconostoc citreum* CBA3623

**DOI:** 10.3389/fmicb.2026.1833544

**Published:** 2026-06-03

**Authors:** Wenchao Zhang, Xingyue Wang, Jinsong Liu, Shiyong Huang, Liyan Yang, Juan Li, Maochun Wei, Zhuangjian Qin, Dengfeng Yang, Lixia Pan

**Affiliations:** 1 State Key Laboratory of Non-Food Biomass Energy Technology, Guangxi Key Laboratory of Marine Natural Products and Combinatorial Biosynthesis Chemistry, Institute of Biology, Guangxi Academy of Marine Sciences, Guangxi Academy of Sciences, Nanning, China; 2 Guangzhou Institutes of Biomedicine and Health, Chinese Academy of Sciences, Guangzhou, China; 3 School of Mathematics and Statistics, Guilin University of Technology, Guilin, China; 4 Guangxi Research Institute of Chemical Industry Co., Ltd., Nanning, China

**Keywords:** dextran, dextransucrase, enzyme engineering, medium molecular weight, molecular-weight distribution

## Abstract

The dextransucrase DarM from *Leuconostoc citreum* CBA3623, a large GH70 family enzyme of approximately 213 kDa, was refractory to full-length expression in *E. coli*. Based on the predicted domain architecture, two catalytically active truncation variants-DarM-ΔV1 (146 kDa) and DarM-ΔV2 (124 kDa), were rationally designed and expressed in soluble form. Both variants exhibited optimal activity under acidic conditions (pH 4.5) but differed in their temperature optima and kinetic parameters. Although these variants produced glucans from high-molecular-weight (HMW) dextran to oligosaccharides, the molecular weight distribution was highly dependent on reaction conditions. Notably, DarM-ΔV1 uniquely enabled one-step synthesis of a medium-molecular-weight (MMW) dextran fraction (~45 kDa). *In silico* structural modeling and site-directed mutagenesis suggested that a putative sugar-binding pocket (V-C) located at the C-terminal region may contribute to the chain length control during dextran synthesis. Disruption of this pocket via the Y1283A mutation impaired MMW dextran synthesis and shifted the product distribution toward oligosaccharides. Guided by this structural insight, we further tuned the reaction temperature (from 35 °C to 5 °C) to expand its accessible MMW window to 27.8–75.2 kDa. Collectively, this work identifies DarM-ΔV1 as a practical and convenient biocatalyst scaffold for MMW dextran production and provides clues to the catalytic mechanism underlying controlled dextran synthesis in GH70 dextransucrase.

## 1 Introduction

Dextran is a water-soluble, *α*-glucan homopolysaccharide that predominantly composed of *α*-(1,6) glycosidic linkages (>50%) with minor branches involving *α*-(1,4), *α*-(1,3) or *α*-(1,2) linkages (Díaz-Montes, 2021). Owing to its satisfactory chemical stability, hydrophilicity, biodegradability, and excellent biocompatibility, dextran finds broad applications in the food, cosmetic and biomedical industries with significant commercial value (Li et al., 2020). Importantly, molecular weight (MW) is one of the key determinants of dextran’s physicochemical properties and application scenarios; dextrans in the medium-molecular-weight (MMW) range, especially around 40–70 kDa, is of substantial pharmaceutical and commercial value (Lanvers et al., 2025). For instance, dextran around 69 kDa has been used as a cytoprotectant to avoid damage during the freezing and thawing treatment in cell preservation (Kuleshova et al., 2001), whereas dextran at 70 kDa with a low degree of branching has been widely used as a blood volume expander, and dextran at 40 kDa has been used to inhibit erythrocyte aggregation and improve the blood flow (Bark and Grände, 2014). Consistent with their established clinical relevance, Dextran 40 and Dextran 70 are listed in the United States Pharmacopoeia and Chinese Pharmacopoeia for injectable use. Moreover, dextran of 40 kDa can also function as a drug delivery system for the treatment of osteoarthritis and has been used in RNA interference (RNAi)-based gene therapy (Chen et al., 2019a; Sheng et al., 2025).

Despite its increasing demand and high-quality requirements, dextran is currently produced mainly through the conventional microbial fermentation, subsequent acid hydrolysis and extensive downstream purification processes are required to obtain the specific MW dextran fractions (Iqbal et al., 2017). This is mainly because the dextran naturally produced by most lactic acid bacteria (LAB) generally falls in the high-MW range (171–441 MDa), typically far exceeding the MW window of many biomedical applications (Zarour et al., 2017). Despite its cost-effectiveness, the acid hydrolysate often contains a wide range of dextran sizes due to the unspecific hydrolyzation reaction, leading to low yields and raw material waste. Since the dextran synthesis is catalyzed by extracellular dextransucrases from LAB such as *Leuconostoc*, *Weissella*, *Lactobacillus* or *Streptococcus*, using sucrose as the substrate (Díaz-Montes, 2021), direct one-step enzymatic synthesis of MMW dextran in a controlled enzymatic manner could offer a simpler and efficient route that reduces downstream processing and product loss.

Dextransucrase (EC 2.4.1.5) is a group of glucansucrase (GS) belonging to the glycoside hydrolase family 70 (GH70), which are large enzymes typically ranging from 150 to 200 kDa. Their catalytic properties and glucan products vary substantially because of differences in domain architecture and catalytic behavior. Dextransucrases purified directly from LAB culture supernatants often suffer from activity loss, low purification efficiency, and contamination by co-produced dextran (Lanvers et al., 2025). Therefore, heterologous expression from various *Escherichia coli* (*E. coli*) strains has become a practical strategy. From a microbiotechnology perspective, heterologous expression also provides a tractable platform for rational engineering of dextransucrases, enabling targeted modulation of their enzymatic properties and product characteristics.

Importantly, structural studies on GH70 enzymes have begun to reveal molecular determinants involved in dextran polymerization and chain-length control. Several truncated GH70 glucansucrases share a common five-discontinuous-domain organization (V-IV-B-A-C-A-B-IV-V) with a global U-shaped fold (Vujicic-Zagar et al., 2010). In particular, sugar-binding pockets in domain V that located at the N- and C-terminal of the enzymes, have been implicated in glucan-chain elongation and retention. Truncation of domain V in GTF180 heavily impairs its dextran synthesis ability (Meng et al., 2015), while mutations in proximal pockets of DSR-OK drastically reduced dextran MW from > 10^9^Da to 10–13 kDa (Claverie et al., 2020). N-terminal truncations have also been reported to alter activity and shift dextran MW distributions toward lower-MW dextrans (Claverie et al., 2017; Müller and Wefers, 2024). Nevertheless, such modifications typically redirect synthesis from high-MW to low-MW dextrans or oligosaccharides, and an efficient, controllable enzyme tool for the direct production of focused MMW dextran is still limited.

As for MMW dextran production, the coexistence of substantial HMW or LMW fractions lead to downstream fractionation, thereby diminishing the practical value of direct enzymatic synthesis. Previous studies have shown that reaction conditions can affect dextran size, whereas this condition-dependent control is not universal and often fails to eliminate high-MW fractions completely. Collectively, these limitations indicate that controlled one-step enzymatic synthesis of a relatively focused MMW dextran remains challenging. In addition, the structural basis underlying size control across different dextransucrases remains incompletely understood. Therefore, there is an urgent need to investigate additional dextransucrases with well-characterized mechanisms that enable the one-step synthesis of homogeneous dextrans at defined MWs, particularly the clinical-type dextrans (40–70 kDa) (Lanvers et al., 2025).

In this study, we identified a dextransucrase gene (*darM*) from *Leuconostoc citreum* CBA3623 and engineered two truncation variants (DarM-ΔV1 and DarM-ΔV2) to enable soluble expression in *E. coli* while retaining their catalytic function. We systematically characterized their enzymatic properties and dextran MW distributions. Notably, DarM-ΔV1 showed a distinctive enzyme-loading dependence enabling one-step enrichment of a focused MMW dextran fraction at ~45 kDa. This was achieved under simple reaction format without the need for external acceptors or enzyme immobilization, highlighting an intrinsic catalytic feature of this engineered enzyme scaffold. We further explored its structural features potentially associated with this phenotype and identified a C-terminal putative sugar-binding pocket (V-C) associated with chain-length control. Furthermore, we also evaluated reaction temperature as a tunable parameter and found that the accessible MMW window could be expanded to approximately 75 kDa. Together, this work identifies DarM-ΔV1 as a promising scaffold for the one-step production of tunable MMW dextran and provides mechanistic clues to support future engineering of GH70 dextransucrases.

## 2 Materials and methods

### 2.1 Sequence analysis of the *darM* gene

The *L. citreum* strain CBA3623 was originally isolated from kimchi (GenBank accession no. NZ_CP042398.1) and its dextransucrase encoding gene *darM* (GenBank accession no. PV818125), was obtained via gene synthesis (Genecreate, China). The signal peptide of the darM gene was predicted using SignalP (Version 6.0).^1^

For phylogenetic analysis, the deduced amino-acid sequence of *darM* (DarM) was aligned with representative glucansucrases (Supplementary Table S1) using the Clustal Omega server.^2^

**Figure 1:**
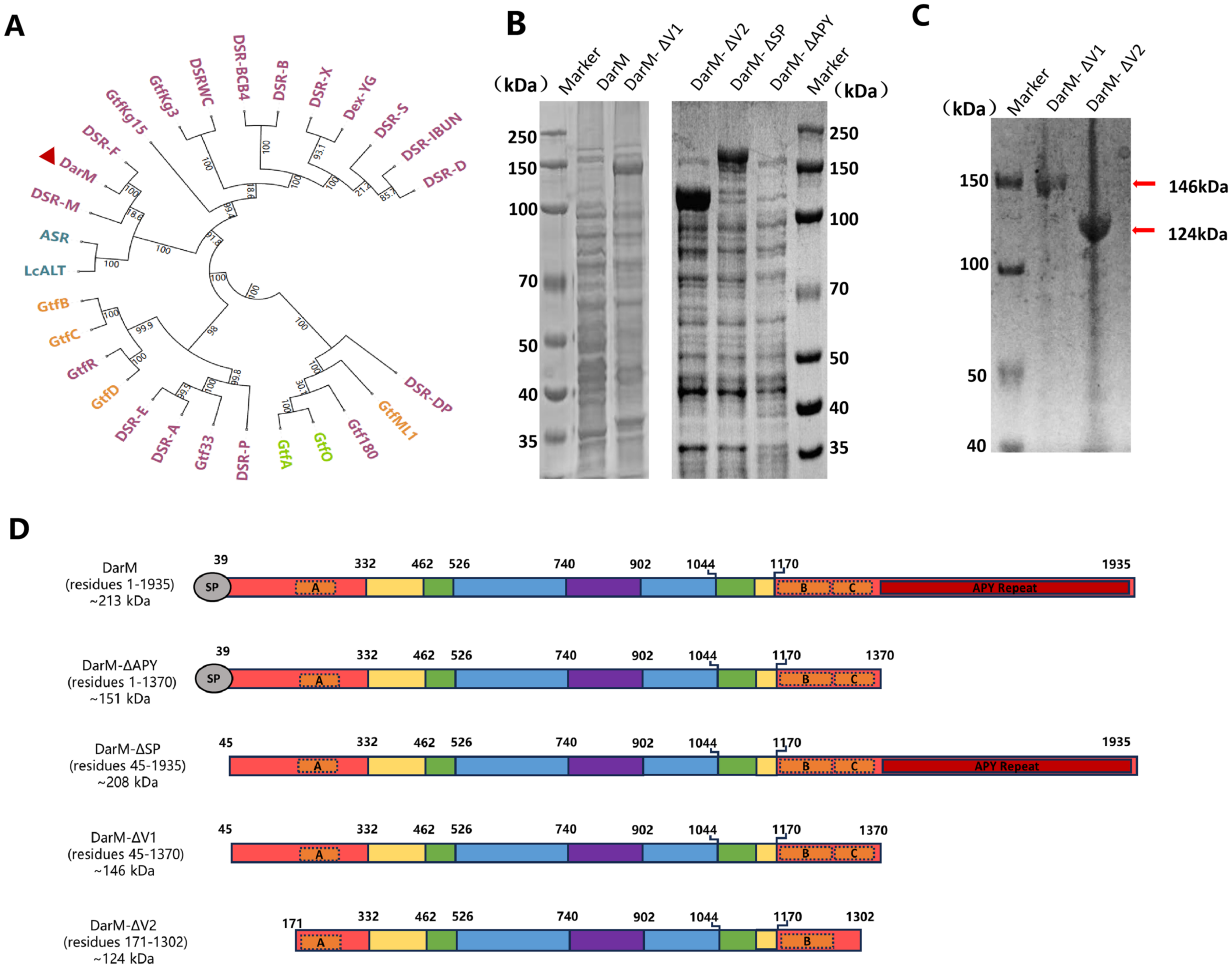
**(A)** Phylogenetic analysis of DarM and representative glucansucrases (GSs). Dextransucrase, mutansucrase, alternansucrase, and reuteransucrase are shown in purple, yellow, blue, and green, respectively. DarM is indicated by a red triangle; **(B)** SDS-PAGE analysis of recombinant proteins expressed in *E. coli*. Recombinant DarM and its truncated variants were induced with 0.25 mM IPTG at 16 °C for 20 h, and the soluble fractions of lysates were analyzed by SDS-PAGE; **(C)** SDS-PAGE analysis of purified DarM-ΔV1 and DarM-ΔV2 proteins. The expected molecular masses of DarM-ΔV1 and DarM-ΔV2 are ~146 and ~124 kDa, respectively; **(D)** schematic structural organization of DarM and its truncated variants, based on amino acid alignment and structural analysis. The signal peptide is shown in gray; domain V in red; domain IV in yellow; domain A in blue; domain B in green; and domain C in purple. Orange dashed boxes indicate putative sugar-binding pockets, and the dark red boxed region indicates APY repeats.

Truncation boundaries of DarM were determined by alignment with structurally characterized GH70 enzymes (Figure 1D).

### 2.2 Construction of recombinant plasmids and *E. coli* expression

DNA fragments were amplified using the primer pairs listed in Supplementary Table S2 and cloned into the pET-28a(+) vector (Sangon Biotech, Shanghai, China). All recombinant plasmids were verified by Sanger sequencing. The BL21(DE3) *E. coli* competent cells were transformed with the recombinant plasmids, and single colonies were incubated in Luria-Bertani (LB) medium (10 g/L tryptone, 5 g/L yeast extract, and 10 g/L NaCl) supplemented with 100 μg/mL kanamycin at 37 °C until the OD_600_ reached 0.6–0.8. Protein expression was then induced by 0.25 mM isopropyl-*β*-D-1-thiogalactopyranoside (IPTG) for 20 h at 16 °C.

Cells were collected and disrupted by ultrasonication, and the supernatant was obtained after centrifugation (12,000 × g, 30 min, 4 °C). These recombinant proteins, which carried an N-terminal 6 × His tag, were purified by using Ni^2+^-NTA agarose (Beyotime, Shanghai, China) and analyzed by SDS-PAGE followed by Coomassie blue staining.

### 2.3 Enzyme activity assay

Dextransucrase activity was assayed using the DNS method (Miller, 1959). Reducing sugars were quantified as fructose equivalents based on a fructose standard curve.

Unless otherwise stated, standard reactions conditions were as follows: a final volume of 1.0 mL calcium acetate buffer (50 mM, pH 5.4) containing 100 mM sucrose, incubated at 25 °C for 10 min. Inactivated enzyme was used as blank control. One unit (U) of enzyme activity was defined as the amount of enzyme that released 1 μmol of reducing sugars (expressed as fructose equivalents) per minute under the above conditions.

### 2.4 Influences of pH and temperature and determination of kinetic parameters

The pH optimum was determined at 25 °C for 10 min in 50 mM buffer containing 100 mM sucrose and 0.01 mg/mL purified enzyme, using calcium acetate buffer (pH 4.0–6.0) and sodium phosphate buffer (pH 6.0–7.0). pH stability was assessed by pre-incubating the purified enzymes in the above buffers at 4 °C for 1 h, followed by measuring residual activity under standard conditions. The optimal temperature was determined by assaying enzyme activity at 20–60 °C (5 °C increments). Thermal stability was assessed by pre-incubating enzyme solutions at 20–60 °C for 1 h, and then measuring residual activity under standard conditions.

Kinetic parameters were determined via the DNS assay using sucrose at different concentration (0–800 mM) with purified enzyme (0.005 mg/mL) under their optimal pH and temperature conditions. *K_m_* and *V_max_* values were obtained after nonlinear regression fitting to the Michaelis–Menten equation using GraphPad Prism 9 (mean ± SD, *n* = 3).

### 2.5 Dextran synthesis and characterization

Unless otherwise stated, polysaccharide-synthesis reactions were performed in a final volume of 1.0 mL in 50 mM calcium acetate buffer (pH 5.4) containing sucrose as the substrate and purified enzyme at the indicated concentration. Although the enzymes exhibited high activity at pH 4.0–5.0 in the DNS assay, slight background signals were also detected. Therefore, pH 5.4 was chosen to minimize potential interference. Moreover, reactions were generally conducted at a mild temperature (25 °C) for long-term incubations. Reactions were terminated by heating at 100 °C for 5 min, followed by centrifugation (12,000 × g, 10 min) to remove insoluble materials. The supernatants were filtered through a 0.22 μm syringe filter prior to chromatographic analysis.

Reaction products were analyzed using a Dionex Ultimate 3000 high-performance liquid chromatography (HPLC) system equipped with a refractive index detector (RID) and a TOSOH TSK G3000 column (7.8 × 300 mm). The column temperature was maintained at 60 °C, and ddH₂O was used as the mobile phase at a flow rate of 0.6 mL/min. Samples and dextran standards were dissolved in ddH₂O at 10 mg/mL, and 10 μL each sample was injected for analysis. Elution profiles were interpreted based on retention times and compared with commercial dextran standards (T5, T20, T50, T100, and T400; Sigma-Aldrich).

Apparent MW distributions of the synthesized dextran were determined by aqueous high-performance gel permeation chromatography (HPGPC) using a Shodex OHpak SB-806 M HQ column coupled to a RID-20A refractive index detector and eluted with 0.1 M NaNO₃.

### 2.6 Dextran purification, quantification and NMR analysis

As described elsewhere (Chen et al., 2019b), the reaction products were first treated by the Sevag method and then purified by three rounds of ethanol-precipitation. The purified polysaccharides were freeze-dried to constant weight. For quantitative analysis, the lyophilized dextran was weighed for gravimetric determination of absolute yield, and the corresponding apparent conversion value was calculated from the recovered polymer mass relative to the theoretical glucosyl mass provided by sucrose (Müller and Wefers, 2024). For NMR analysis, 30 mg of lyophilized dextran was dissolved in 500 μL deuterium oxide (D₂O) and analyzed by 800 MHz ^1^H NMR spectroscopy, with chemical shifts referenced to residual half-heavy water (HDO) at *δ* 4.80 ppm (30 °C). All spectra were processed using MestreNova 5.3.1 (Mestrelab Research). Linkage types and their relative proportions were assigned based on the chemical shifts (*δ*) and integrated signal intensities of the anomeric proton signals (Fabre et al., 2005; Chen et al., 2019b).

### 2.7 Molecular dynamics simulations

Molecular dynamics (MD) simulations were performed using AMBER 22. The protein was parameterized with the ff14SB force field, and the ligand parameters were generated via AmberTools 23 (Maier et al., 2015). The protein-ligand complex was solvated in an octahedral box with TIP3P water molecules, and the system was neutralized using Na^+^ ions. Energy minimization was carried out using the steepest descent method, followed by equilibration through 100 ps of NVT ensemble and 200 ps of NPT ensemble. A 50 ns production MD run was then performed at 300 K and trajectory analyses were carried out using cpptraj (included in AmberTools23).

## 3 Results and discussion

### 3.1 Design, construction and expression of truncated variants

The full-length *darM* gene comprises an open reading frame of 5,808 bp and encodes a 1,936-residue protein (~213 kDa). Phylogenetic analysis clusters DarM within the dextransucrase clade (Figure 1A), sharing close relationship to DSR-F from *L. citreum* B/110-1-2 (98.9% sequence identity) (Fraga Vidal et al., 2011) and the well-characterized DSR-M from *L. citreum* NRRL B-1299 (67.8% identity) (Passerini et al., 2015), respectively. Domain annotation, based on sequence features and structural alignment with the homologs GTF180 (Vujicic-Zagar et al., 2010), DSR-M (Claverie et al., 2019) and ASR (Molina et al., 2019), showed that DarM displays a typical GH70 architecture, which is comprised of five non-continuous domains that form a U-shaped overall fold. An N-terminal signal peptide (SP) and a C-terminal APY-repeat region were found in domain V (Figure 1D).

Because the full-length DarM (residues 1–1,935, ~213 kDa) failed to express in *E. coli* (Figure 1B), four truncation variants were rationally designed. A previous report found that APY repeats are non-essential for the catalytic activity of GSs (Moulis et al., 2006); so this region was first truncated to generate DarM-ΔAPY (residues 1–1,370, ~150 kDa), which still showed no obvious expression (Figure 1B). In contrast, when the SP was truncated (DarM-ΔSP, residues 45–1,935, ~208 kDa), an expected band was observed the soluble fraction (Figure 1B), although the purification yield remained low. The SP was predicted to be a Sec/SPI-type signal associated with host secretion; retention of this domain in the expressed recombinant protein may affect soluble expression in *E. coli* (Kleiner-Grote et al., 2018). Further deletion of both SP and the APY-repeat (DarM-ΔV1, residues 45–1,370, ~146 kDa) markedly improved soluble expression (Figure 1B). To additionally remove non-conserved linker segments, DarM-ΔV2 (residues 171–1,302, ~124 kDa) was constructed and showed further improvement in soluble expression (Figure 1B). Both truncates were successfully purified by Ni^2+^-NTA affinity chromatography (Figure 1C), with recovery rate of 47.2 and 62.7%, corresponding to purification folds of 54.6 and 62.6, respectively. The specific activity of purified DarM-ΔV1 and DarM-ΔV2 were determined to be 14.7 U/mg and 15.8 U/mg, respectively.

### 3.2 Enzymatic characterization of DarM variants

**Figure 2:**
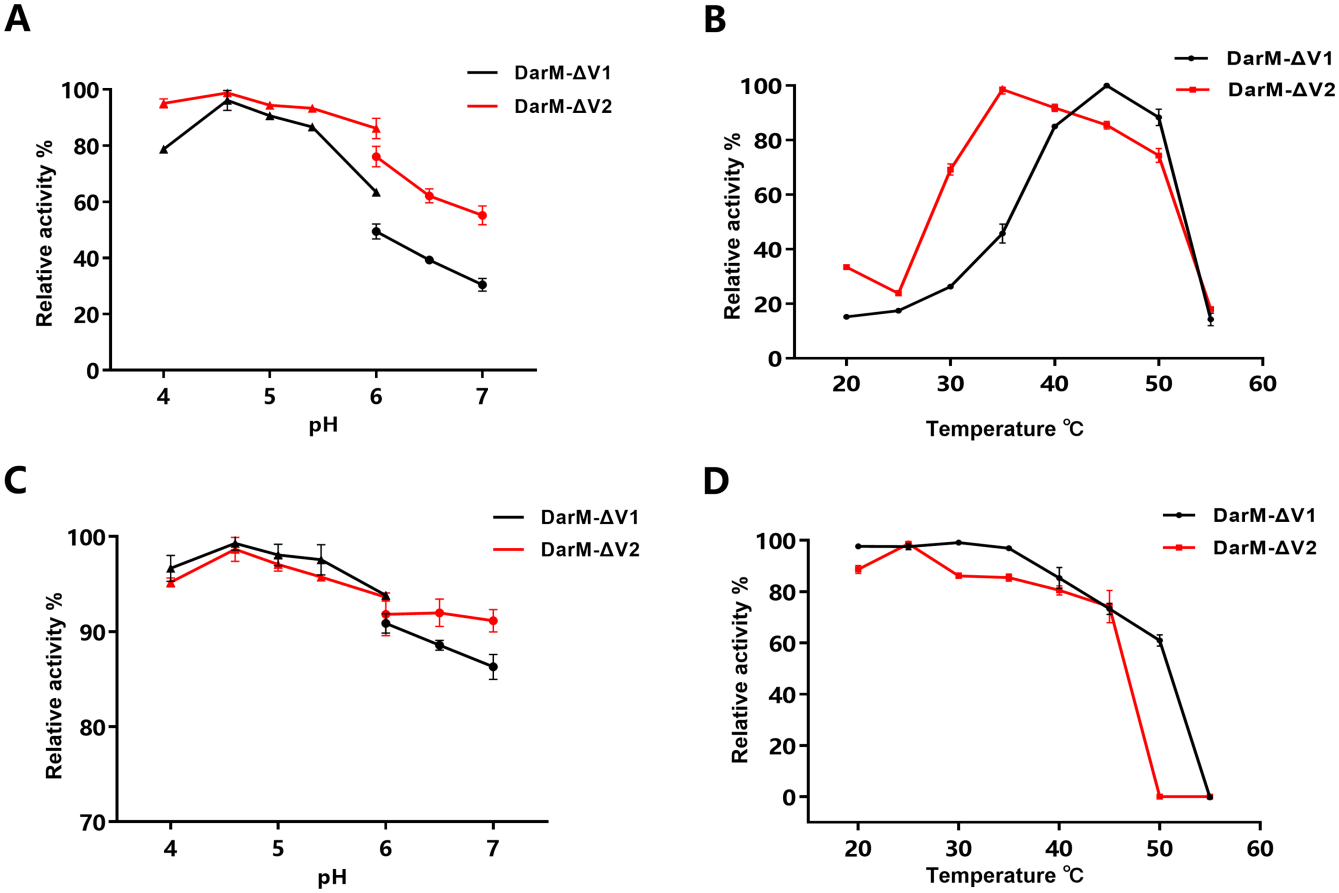
Enzymatic characterization of recombinant dextransucrase variants DarM-ΔV1 and DarM-ΔV2. **(A)** The pH optimum of DarM variants. Enzyme activity was determined at 25 °C across a pH range of 4.0–7.0 using calcium acetate buffer (pH 4.0–6.0) and sodium phosphate buffer (pH 6.0–7.0); **(B)** the optimal temperature of DarM variants. Enzyme activity was determined at pH 5.4 across a temperature range of 20–60 °C; **(C)** the pH stability of DarM variants. Enzymes were pre-incubated in buffers at the indicated pH for 1 h at 4 °C, and residual activity was measured under standard conditions; **(D)** the thermal stability of variants. Enzymes were pre-incubated at the indicated temperatures for 1 h, and residual activity was measured under standard conditions. Relative activity was calculated by setting the highest activity of each variant in each assay to 100%. Data are presented as mean ± SD from three independent experiments.

As shown in Figure 2A, both variants displayed maximal activity at an acidic condition (pH 4.5), which is similar to several reported dextransucrases such as LcDS from *L. citreum* HJ-P4 (pH 5.5) (Yi et al., 2009), Gtf-DSM from *Lactobacillus ingluviei* DSM 14792 (pH 4.5) (Chen et al., 2019b), the *Liquorilactobacillus nagelii* dextransucrase variant dsr3510ΔC-term (pH 4.5) (Bechtner et al., 2022), ΔN190*Lm*DexA from *Leuconostoc mesenteroides* NN710 (pH 5.6) (Zuo et al., 2024), and DsrB from *L. citreum* JZ-002 (pH 5.0) (Pu et al., 2024). For pH stability, both enzymes retained more than 90% of their initial activities at pH 4.0–6.0, and the residual activities remained above 85% at pH 6.5–7.0 (Figure 2C), indicating good pH stability.

The optimal reaction temperatures of DarM-ΔV1 and DarM-ΔV2 were determined of as 45 °C and 35 °C, respectively (Figure 2B). Reported dextransucrases commonly display optimal temperatures around 30–35 °C, such as 35 °C for LcDS (Yi et al., 2009) and Gtf-DSM (Chen et al., 2019b), and 30 °C for ΔN190*Lm*DexA and DsrB (Pu et al., 2024; Zuo et al., 2024). Compared to DarM-ΔV1, DarM-ΔV2 showed a comparatively decreased optimum temperature, which resembled to the *L. nagelii* dextransucrase system, where the optimum temperature decreased from 40 °C (full-length dsr3510) to 25 °C (truncated dsr3510ΔC-term) (Bechtner et al., 2022). In the thermostability assay, both variants retained more than 75% activities after preincubation at 20–45 °C for 1 h. DarM-ΔV1 showed higher thermotolerance than DarM-ΔV2, since it retained approximately 60% activity at 50 °C under which the DarM-ΔV2 become nearly inactive (Figure 2D). Overall, the thermostability of reported dextransucrases is generally not satisfying. For example, the residual activity of ΔN190LmDexA decreased below 40% after incubation at 35 °C for 1 h (Zuo et al., 2024), whereas Gtf-DSM lost its activity rapidly at 50 °C (Chen et al., 2019b). In special, DarM-ΔV1 retained more than 60% of its activity after preincubation at 50 °C for 1 h, indicating good application prospect.

Based on the results above, kinetic parameters of the variants were further determined under their optimal conditions (Supplementary Figure S1). The *K_m_* of DarM-ΔV2 (43.19 ± 0.78 mM) was higher than that of DarM-ΔV1 (32.60 ± 0.29 mM), indicating a lower apparent affinity for sucrose under these reaction conditions. In contrast, DarM-ΔV2 exhibited a higher apparent *V_max_* than DarM-ΔV1, increasing from 25.78 ± 0.19 to 36.24 ± 0.44 μmol·min^−1^·mg^−1^.

### 3.3 Reaction conditions modulate dextran MW distribution

To investigate their catalytic product profiles, the total reaction products generated by DarM variants were analyzed by HPLC under varied reaction time, sucrose concentration, and enzyme concentration. In this work, based on dextran standards, the chromatograms were divided into HMW (>100 kDa), MMW (20–100 kDa), LMW (5–20 kDa), and oligosaccharide/monosaccharide regions (Supplementary Figure S2).

#### 3.3.1 Effect of reaction time

**Figure 3:**
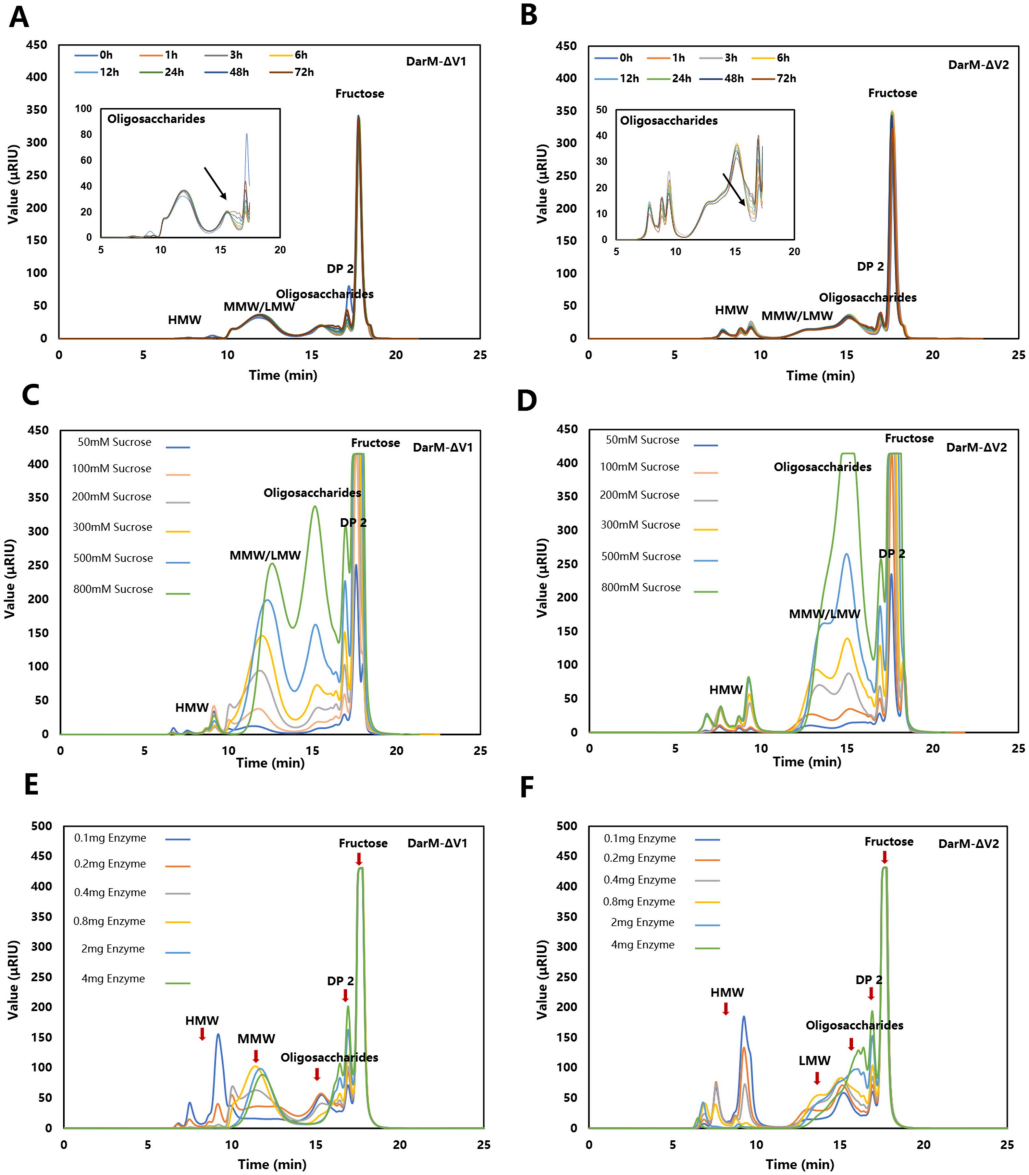
Representative HPLC chromatograms of products generated by DarM variants under different reaction conditions. **(A,B)** Reactions containing 100 mM sucrose and 0.4 mg/mL enzyme were quenched immediately after mixing (0 h) or after incubation for up to 72 h; **(C,D)** effect of sucrose concentration. Reactions were performed for 12 h with 0.4 mg/mL enzyme and the indicated sucrose concentrations (50–800 mM); **(E,F)** effect of enzyme concentration. Reactions were performed for 12 h with 200 mM sucrose and the indicated final enzyme concentrations (0.1–4 mg/mL).

Reactions were performed at a fixed final enzyme concentration of 0.4 mg/mL and sucrose concentration of 100 mM. The 0 h sample was obtained by quenching the reaction immediately after enzyme addition, whereas the other samples were incubated from 1 to 72 h. As showed in Figures 3A,B, the polymer size was fixed at the earliest sampled time point and remained largely unchanged thereafter, indicating rapid initial glucan formation, and byproduct fraction was not detected in the no-enzyme control (Supplementary Figure S3). Unlike the HMW-dextran synthesize enzymes such as DSR-OK and DSR-S, which were characterized by the time-dependent increase of HMW content (Moulis et al., 2006; Claverie et al., 2020; Ma et al., 2025), DarM truncations do not show such time-dependent elongation and accumulation pattern (Figures 3A,B). In contrast, the polymer content remained stable or showed a slight decrease after prolonged incubation, an effect that was more pronounced at an increased enzyme concentration (2 mg/mL) (Supplementary Figure S4). Meanwhile, the content of oligosaccharide increased progressively throughout the time course (Figures 3A,B; Supplementary Figure S4). This late-stage rise in oligosaccharides has also been reported for GH70 glucansucrase systems upon sucrose depletion, which may attributed to accumulating glucosyl transfer competition between small sugars (e.g., glucose, fructose, and leucrose) and dextran (Moulis et al., 2006; Claverie et al., 2020).

#### 3.3.2 Effect of sucrose concentration

In the present study, at a fixed final enzyme concentration of 0.4 mg/mL and a reaction time of 12 h, elevating the sucrose concentration from 50 to 800 mM result in higher contents of all detectable reaction components (Figures 3C,D). In particular, the oligosaccharide region became prominent relative to the MMW/LMW dextran fraction, especially for DarM-ΔV2. Moreover, the MMW/LMW polymer peak seemed to shift toward lower MW as sucrose concentration increased.

The influence of sucrose concentration on GH70 product distributions has been reported previously, although the responses vary substantially. The MW of dextran synthesized by B-512FMC (Kim et al., 2003) and immobilized DsrB (Pu et al., 2024) were reported to increase in higher sucrose, whereas some display the opposite tendency, in which higher sucrose concentrations shift dextran formation toward smaller size and/or oligosaccharides (e.g., B-512FMCM, LaniDSΔN, and SSAL4550) (Falconer et al., 2011; Müller and Wefers, 2025). Similarly, for the DarM variants, increasing sucrose concentration shifted the chromatographic profile toward oligosaccharides and a smaller polymer fraction, suggesting that increasing substrate concentration did not effectively promote continued dextran chain elongation. Tanriseven and Robyt reported that sucrose concentrations above 200 mM promoted the formation of highly branched, LMW dextrans, and proposed that this effect may involve binding to a low-affinity site and altered active-site conformation (Tanriseven and Robyt, 1993). Taken together, 200 mM sucrose was selected as a practical compromise condition for subsequent experiments.

#### 3.3.3 Effect of enzyme concentration

At a fixed reaction condition (200 mM sucrose, 12 h), the enzyme concentration markedly redistributed the dextrans MW. As for DarM-ΔV1, enzyme concentrations at 0.1–0.2 mg/mL yielded HMW dextran fraction together with oligosaccharides; when enzyme concentration increased to 2–4 mg/mL, an enriched MMW dextran fractions were observed (Figure 3E). In contrast, DarM-ΔV2 did not show a comparable shift toward the MMW/LMW fraction; oligosaccharides remained the predominant component at 2–4 mg/mL enzyme (Figure 3F). Collectively, raising enzyme concentration enriched the MMW fraction for DarM-ΔV1, but DarM-ΔV2 remained biased toward LMW products with little enrichment. Enzyme concentration has been less discussed as an independent tuning parameter for glucan’s MW distribution. Falconer’s study on B-512FMC showed that increasing enzyme concentration led to a decrease of dextran MW (Falconer et al., 2011). More recently, a similar trend was also reported: higher immobilized DsrB enzyme concentrations resulted in LMW glucans (Pu et al., 2024). Together, these studies indicate that enzyme concentration can serve as a practical parameter to modulate dextran MW distributions, although the magnitude and direction of the response can vary among enzymes.

### 3.4 Characterization of dextran produced by DarM-ΔV1

**Figure 4:**
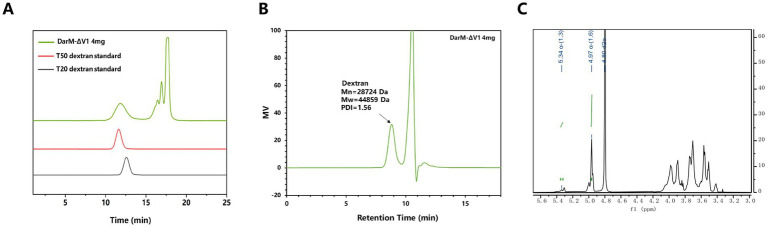
**(A)** HPLC and **(B)** HPGPC chromatograms of dextran produced by 4 mg/mL DarM-ΔV1 and 200 mM sucrose. The corresponding MW and PDI values are indicated; **(C)** ^1^H NMR spectrum of dextran produced by DarM-ΔV1 in D_2_O.

Dextran synthesized under optimized conditions (4 mg enzyme per 1 mL reaction mix, 200 mM sucrose) had an estimated MW of ~45 kDa based on the dextran standards (Figure 4A). HPGPC analysis confirmed an average MW of 44,859 Da with a polydispersity index (PDI) of 1.56, indicating a relatively narrow size distribution (Figure 4B). Based on the lyophilized dry weight, the recovered polymer yield reached ~17.2 g/L, corresponding to an apparent glucosyl conversion of 53.0% into dextran.

The ^1^H NMR spectroscopy results showed that dextrans produced by DarM-ΔV1 containing 86.75% *α*-(1,6) linkages (*δ* 4.97 ppm) together with 13.25% branching via *α*-(1,3) linkages (δ 5.34 ppm) (Figure 4C). Notably, the glucan profile of DarM-ΔV1 differs from the reported DSR-F, despite their high sequence identity (98.9%): the DSR-F variant mainly produces HMW polymers together with DP < 8 oligosaccharides, and its synthesized glucans were reported to be dominated by about 93% *α*-(1,6) linkages with additional *α*-(1,3) and *α*-(1,4) branching (Fraga Vidal et al., 2011). these differences may partly be attributed to truncation design, reaction format, and catalytic conditions, which are highly sensitive for GH70 enzymes.

### 3.5 Structural analysis of DarM-ΔV1 and characterization of putative sugar-binding pockets

**Figure 5:**
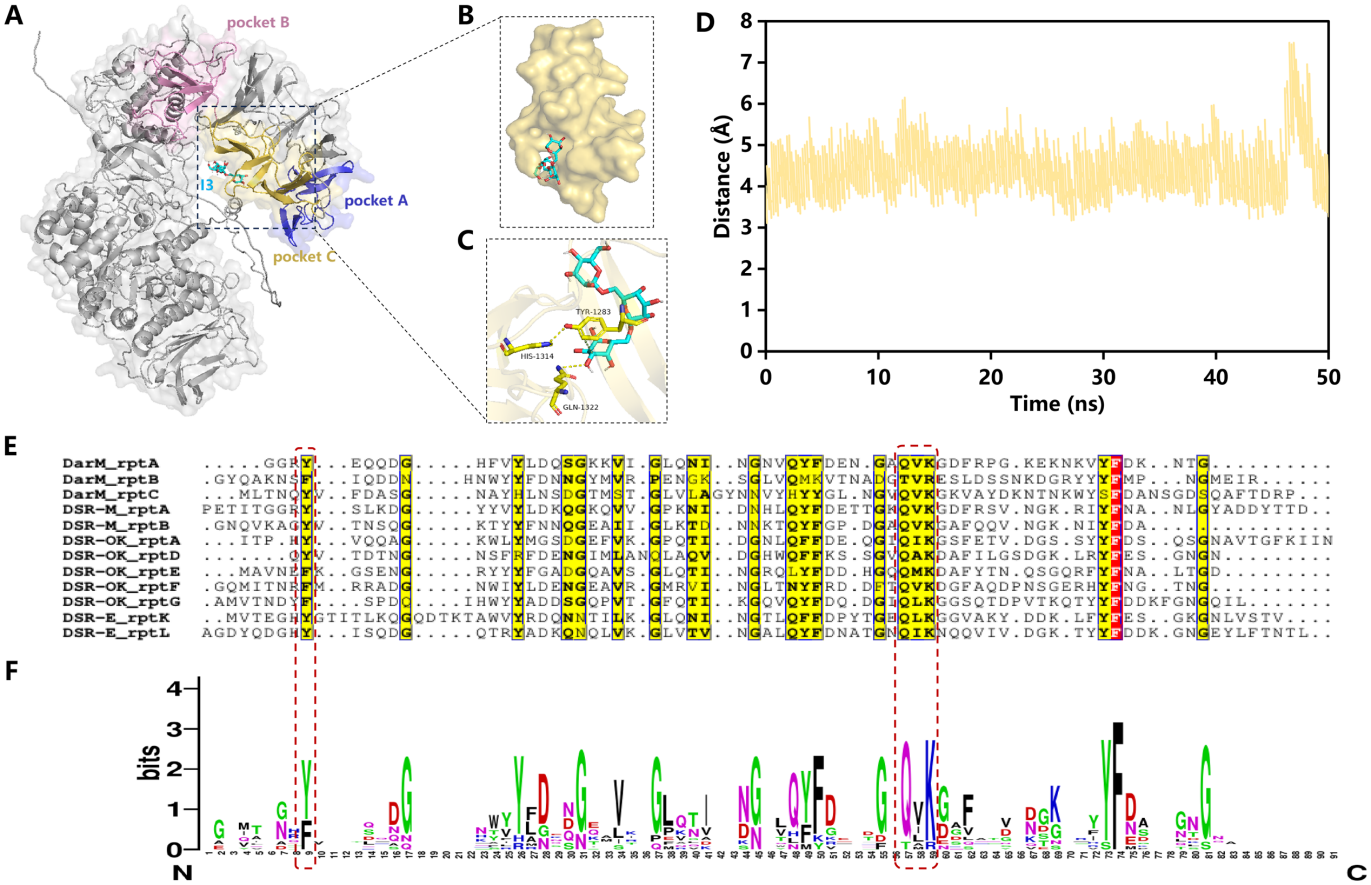
Structural modeling and putative sugar-binding pockets prediction of DarM. **(A)** Structural modeling of DarM highlighting three putative sugar-binding pockets in domain V (V-A, V-B, and V-C), colored blue, pink, and yellow, respectively; **(B)** molecular docking of isomaltotriose with pocket V-C; **(C)** predicted interaction network between isomaltotriose and pocket V-C (dashed lines); **(D)** molecular dynamics simulation analysis of isomaltotriose with pocket V-C, showing the distance fluctuation over 50 ns; **(E)** sequence alignment of DarM pockets with corresponding regions from DSR-E, DSR-M, and DSR-OK, representing putative glucan-binding repeats; **(F)** sequence logo derived from this alignment. The aromatic stacking residue (Tyr/Phe) and the QxK motif are enclosed within a red dashed box.

The structure of DarM-ΔV1 was predicted using AlphaFold and visualized with PyMOL. This model showed high overall confidence, except for the N-terminal region (residues 45–161), which formed an extended loop with low confidence (Figure 5A). Domain V is highly disordered and known to participate in polymer chain elongation in GH70 glucansucrases (Moulis et al., 2006; Meng et al., 2015; Brison et al., 2016; Claverie et al., 2017; Claverie et al., 2019; Molina et al., 2019). Canonical sugar-binding pockets in this domain typically contain an aromatic stacking residue (Tyr/Phe) and a QxK motif (Claverie et al., 2017; Claverie et al., 2020). After sequence alignment with characterized glucansucrases (DSR-M, DSR-OK, and ΔN123-GBD-CD2), we identified three candidate pockets in DarM domain V: V-A (171–231 aa, N-terminal), V-B (1,188–1,258 aa), and V-C (1,278–1,353 aa) (Figures 1D, 5E). V-B retains the aromatic residue but lacks a well-aligned QxK motif, suggesting compromised binding ability (Figures 5E,F).

Previous studies on GH70 enzymes indicate that sugar-binding pockets control polymer size. In DSR-MΔ2, pocket V-A is located near the catalytic cleft but exhibits low binding affinity, resulting in exclusively LMW dextrans via a distributive mechanism (Claverie et al., 2017). In DSR-OKΔ1, disruption of pockets D/E (close to the cleft) markedly reduced processivity and abolished HMM synthesis, while distal pockets (F/G) had minor effects (Claverie et al., 2020). Thus, pockets spatially close to the catalytic cleft exert greater influence on polymer size. In DarM, V-C is positioned closest to the catalytic cleft based on the structural model (Figure 5A). Docking simulations with isomaltotriose (I3) as ligand showed that pocket V-C anchors I3 via hydrogen bonds (His-1314, Gln-1322) and CH–*π* interactions (Tyr-1283), with a predicted binding energy of −6.995 kcal/mol (Figures 5A–C). A 50 ns molecular dynamics simulation confirmed stable association of I3 with V-C, with the Tyr-1283–I3 distance fluctuating around ~4.4 Å (Figure 5D). These results support that pocket V-C is a functional sugar-binding pocket in DarM and may mediate product size control.

### 3.6 Mechanistic insights into dextran size control in DarM-ΔV1

DarM-ΔV1 and DarM-ΔV2 showed markedly different product profiles, with DarM-ΔV2 tending to produce more oligosaccharides (Figures 3C–F), likely due to their distinct N-terminal (residues 45–170) and/or C-terminal (residues 1,303–1,370) regions. Two additional truncation mutants were constructed: DarM-ΔV1ΔN (residues 171–1,370) and DarM-ΔV1ΔC (residues 45–1,302). DarM-ΔV1ΔN displayed a product profile similar to DarM-ΔV1, though with a slightly broader distribution, and DarM-ΔV1ΔC exhibited a heterogeneous product profile (Supplementary Figure S5). These findings suggested that the C-terminal region (residues 1,303–1,370) played an important role in dextran MW distribution.

**Figure 6:**
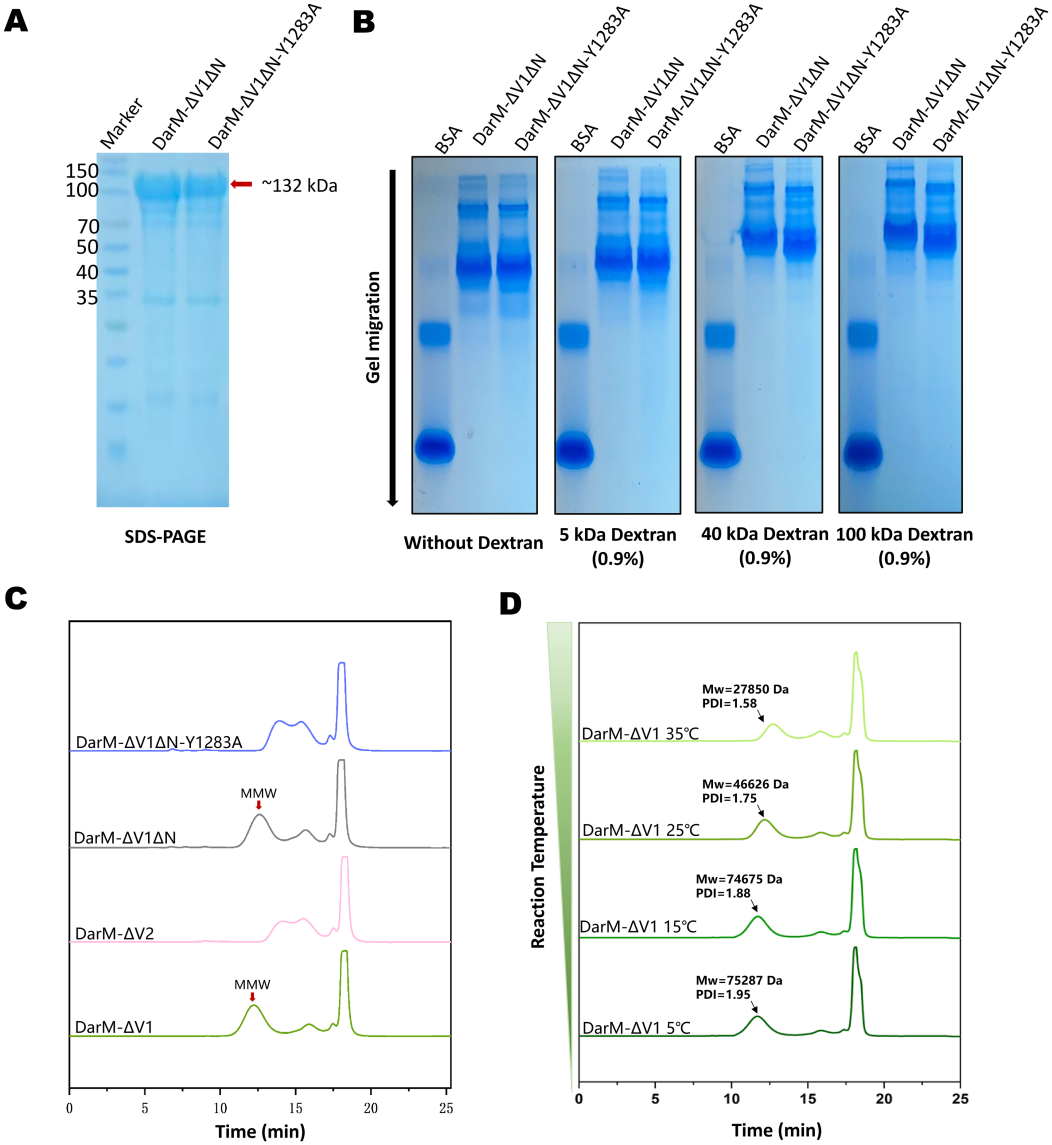
Characterization of the product profiles produced by DarM-ΔV1ΔN and DarM-ΔV1ΔN-Y1283A and affinity gel electrophoresis. **(A)** SDS-PAGE analysis of purified recombinant DarM-ΔV1ΔN and DarM-ΔV1ΔN-Y1283A; **(B)** affinity gel electrophoresis of DarM-ΔV1ΔN and DarM-ΔV1ΔN-Y1283A in gels containing 0.9% dextran of 5 kDa, 40 kDa, and 100 kDa; BSA was included as a reference protein for migration comparison and as a negative control for dextran binding. **(C)** Representative HPLC chromatograms of products synthesized by DarM-ΔV1 (green), DarM-ΔV2 (pink), DarM-ΔV1ΔN (gray), DarM-ΔV1ΔN-Y1283A (blue); **(D)** effect of temperature (5–35 °C) on the product distribution of DarM-ΔV1, analyzed by HPLC. The corresponding MW and PDI values are indicated.

Because this segment overlaps the predicted V-C pocket (1,278–1,353 aa), the site-directed Y1283A mutation was introduced to test whether disrupting this pocket could account for the ΔV1ΔC-associated shift in product profile. The truncates DarM-ΔV1ΔN and DarM-ΔV1ΔN-Y1283A were successfully expressed (Figure 6A) and their catalytic activities were broadly comparable across the tested temperatures. However, under the same reaction conditions (2 mg/mL enzyme, 200 mM sucrose, 25 °C, 10 min), these two variants generated markedly different product profiles (Figure 6C): DarM-ΔV1ΔN produced a dominant MMW dextran fraction, whereas DarM-ΔV1ΔN-Y1283A shifted the profile toward oligosaccharides similar to DarM-ΔV2. To determine whether this shift was caused by their catalytic efficiency, kinetic analysis was performed at 25 °C in a sucrose concentration range of 0–800 mM. DarM-ΔV1ΔN and DarM-ΔV1ΔN-Y1283A showed comparable apparent *V_max_* (34.65 ± 0.66 and 35.53 ± 0.94 μmol·min^−1^·mg^−1^, respectively), and apparent *K_m_* (45.36 ± 1.02 and 44.26 ± 2.50 mM, respectively) values (Supplementary Figure S6), indicating that the single-point mutation did not have significant effect on their kinetic parameters.

Previous studies have shown that sugar-binding pockets in GH70 enzymes can contribute to glucan binding and elongation (Claverie et al., 2020). Compared to DarM-ΔV1ΔN-Y1283A, native PAGE in dextran-containing gels showed stronger retardation for DarM-ΔV1ΔN, whereas the two variants migrated similarly in dextran-free gels (Figure 6B), which support the potential enzyme-dextran interactions via this putative V-C pocket.

Because the putative sugar-binding pocket is hypothesized to contribute to dextran chain-retention/release through noncovalent enzyme-glucan interactions (Claverie et al., 2020), and such interactions can vary with temperature (Jarmoskaite et al., 2020), we further evaluated the potential of temperature as a tunable parameter. As shown in Figure 6D, across 5–35 °C, DarM-ΔV1 maintained a dominant MMW polysaccharide peak, with the dextran size increasing from ~27.8 kDa to ~75.2 kDa. The recovered dextran amounts and corresponding apparent conversion values under these four temperatures varied to some extent, with values of 15.44 g/L (47.7%) at 5 °C, 18.06 g/L (55.74%) at 15 °C, 16.17 g/L (49.9%) at 25 °C, and a lower value of 11.40 g/L (35.2%) at 35 °C, indicating that the temperature-dependent shifts in dextran size did not directly track the recovered dextran amount or the corresponding apparent conversion value. Together, these results indicate that the C-terminal region, especially the putative pocket V-C, underlies DarM-ΔV1’s ability to produce enriched MMW dextran.

## 4 Conclusion

**Figure 7:**
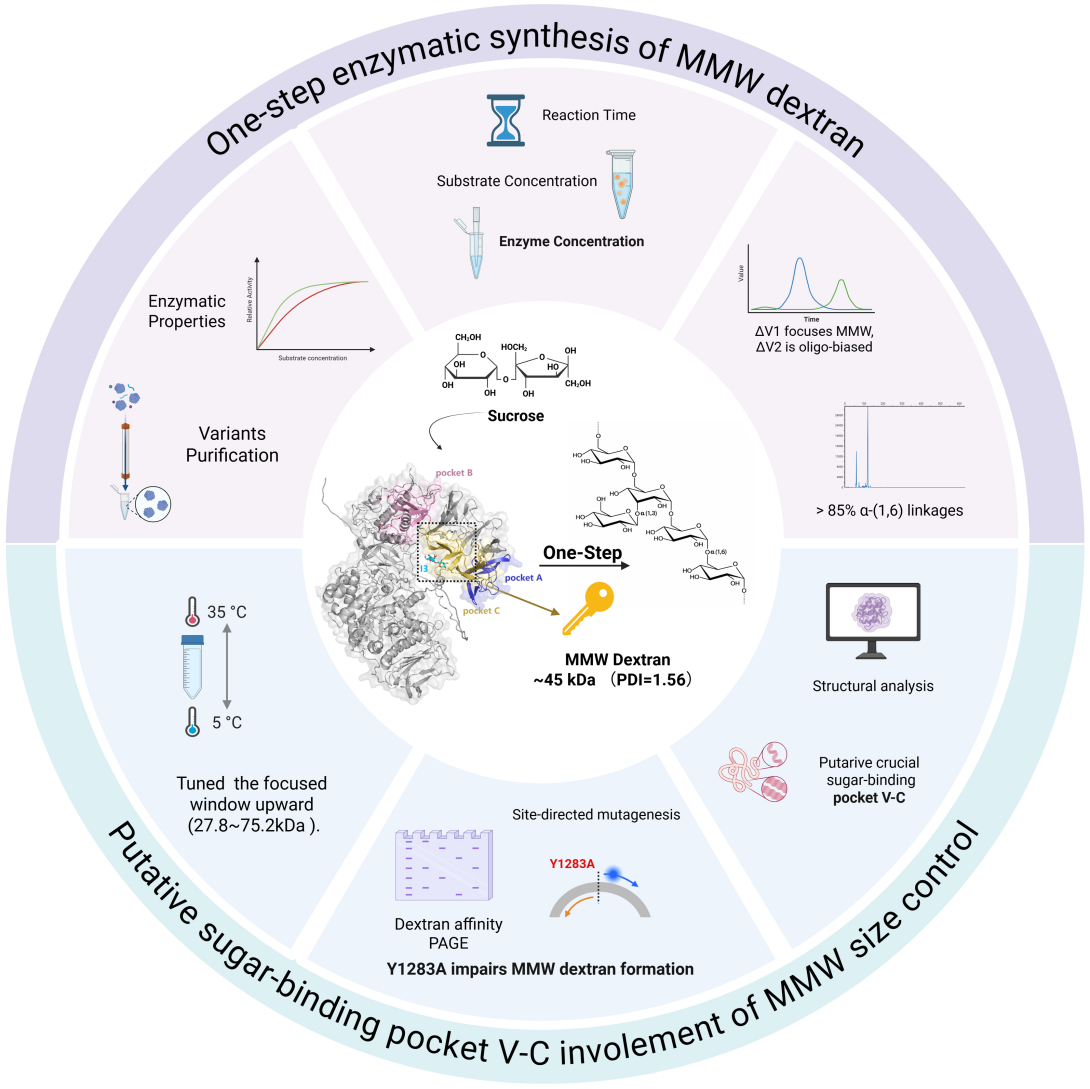
One-step enzymatic synthesis of MMW dextran using engineered dextransucrase variants DarM-ΔV1.

Engineered dextransucrases that enable one-step synthesis of narrowly distributed MMW dextran are scarce. Here, we generated DarM-ΔV1, a truncated GH70 enzyme from *L. citreum*, which produced an enriched MMW dextran fraction (~45 kDa, PDI = 1.56) directly from sucrose. Mechanistic analysis, including truncation mapping and the Y1283A mutation, implicates a C-terminal sugar-binding pocket (V-C) in chain-length control. Promoted by this working model involving chain retention/release, we further introduced temperature as an actionable process parameter. Notably, lowering the reaction temperature shifted the product peak to ~75 kDa, expanding the accessible MMW range via simple parameter adjustment (Figure 7). Together, these findings link structural features with product phenotype and offer a tunable, reproducible strategy for producing defined MMW dextrans.

## Data Availability

The original contributions presented in the study are included in the article/Supplementary material, further inquiries can be directed to the corresponding authors.

## Footnotes

1
https://services.healthtech.dtu.dk/services/SignalP-6.0/

2
http://www.clustal.org/omega/
