# Supplementary material for: One-step enzymatic synthesis of medium molecular weight dextran using engineered dextransucrase DarM from Leuconostoc citreum CBA3623
Source: Front Microbiol. 2026 Jun 3;17:1833544. doi: 10.3389/fmicb.2026.1833544 (PMC13272390; doi:10.3389/fmicb.2026.1833544)
Supplement: Supplementary file 1 [file Data_Sheet_1.zip › Proof-Original Gel images/Proof Revised-Original Gel Image.pdf]

The original gel images

Figure 1B Left

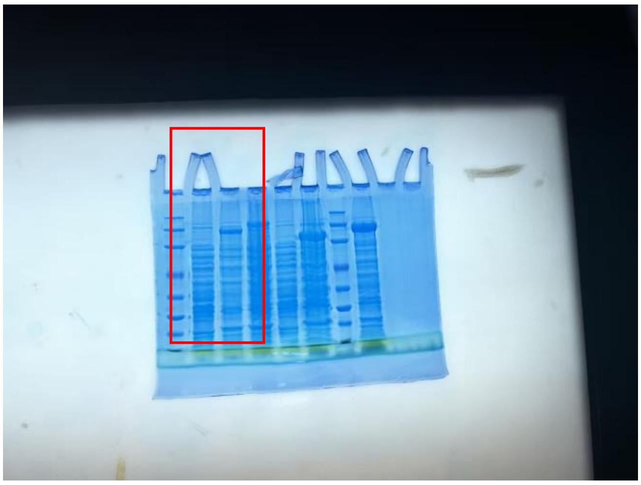

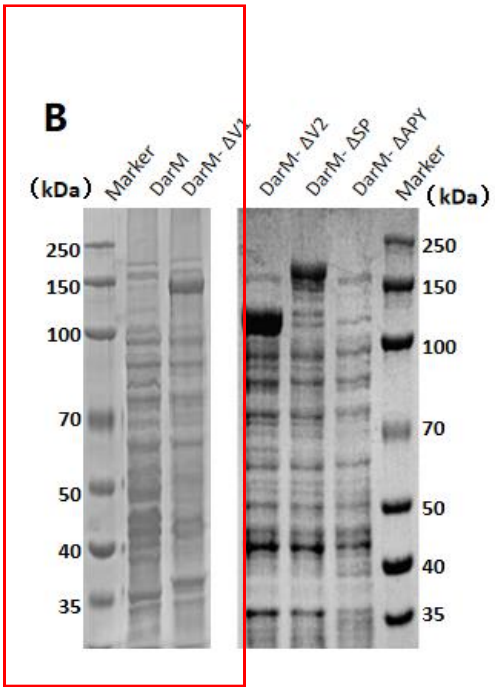

Figure 1B Right

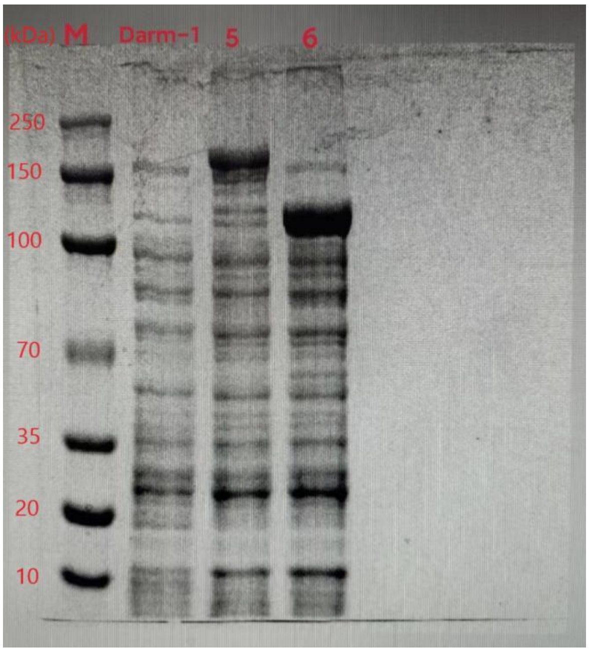

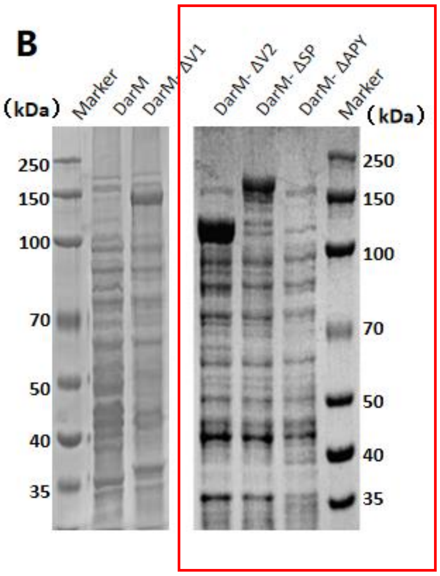

Figure 1C

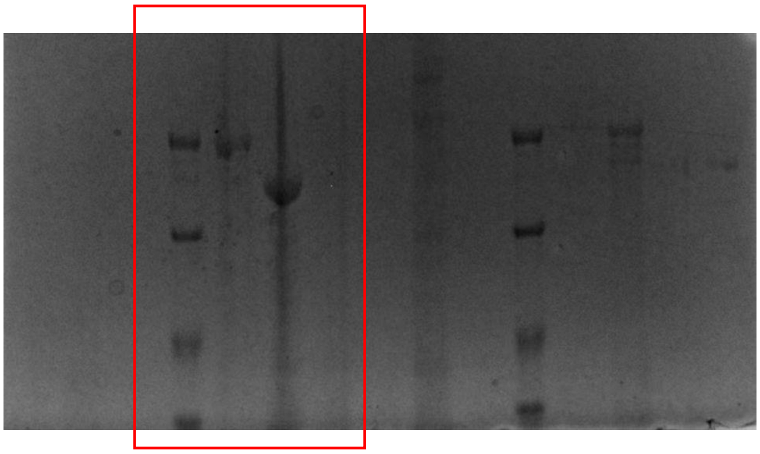

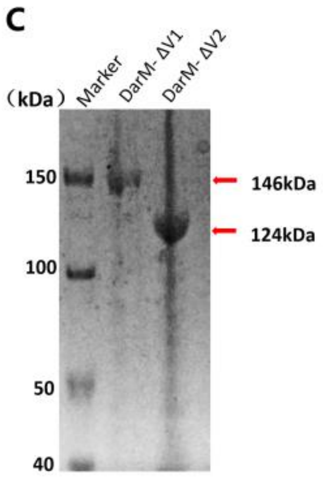

Original Figure 2C -different exposure versions

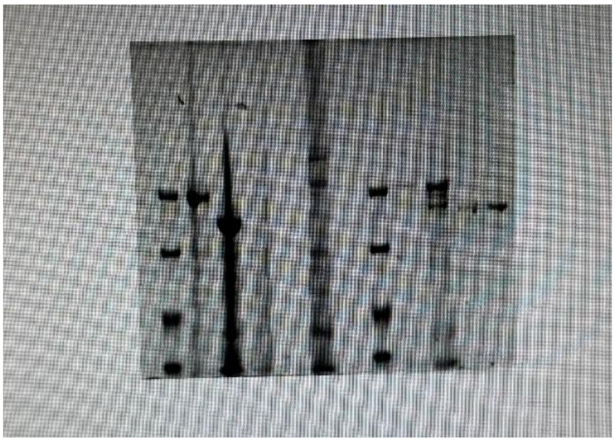

Figure 6A

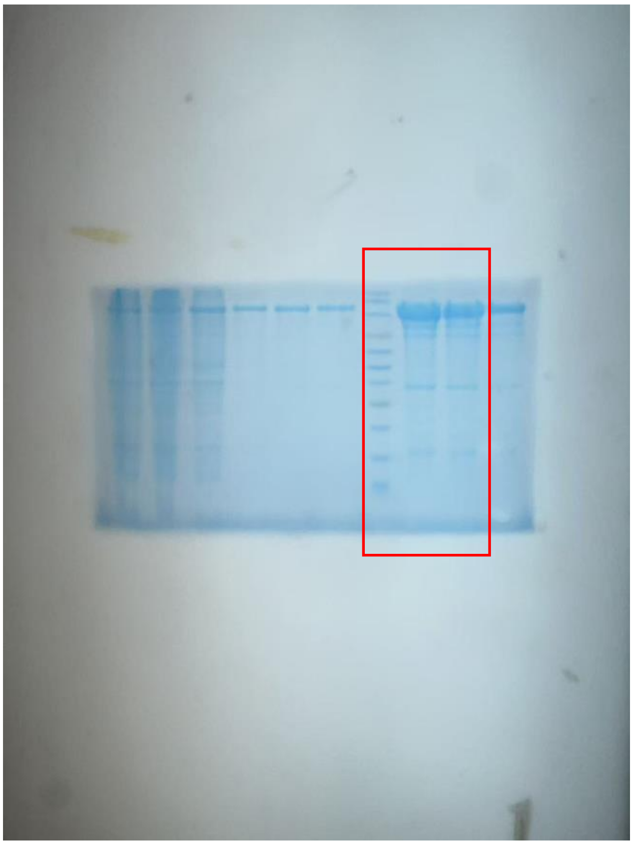

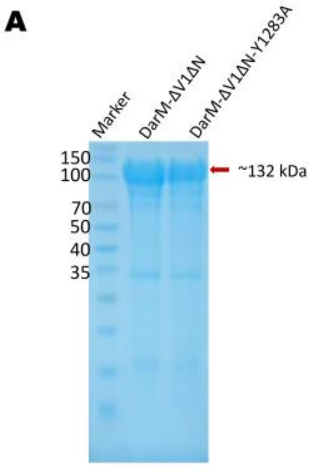
A
Marker
DarM-ΔV1ΔN
DarM-ΔV1ΔN-Y1283A
150
100
70
50
40
35
~132 kDa
SDS-PAGE

Figure 6B

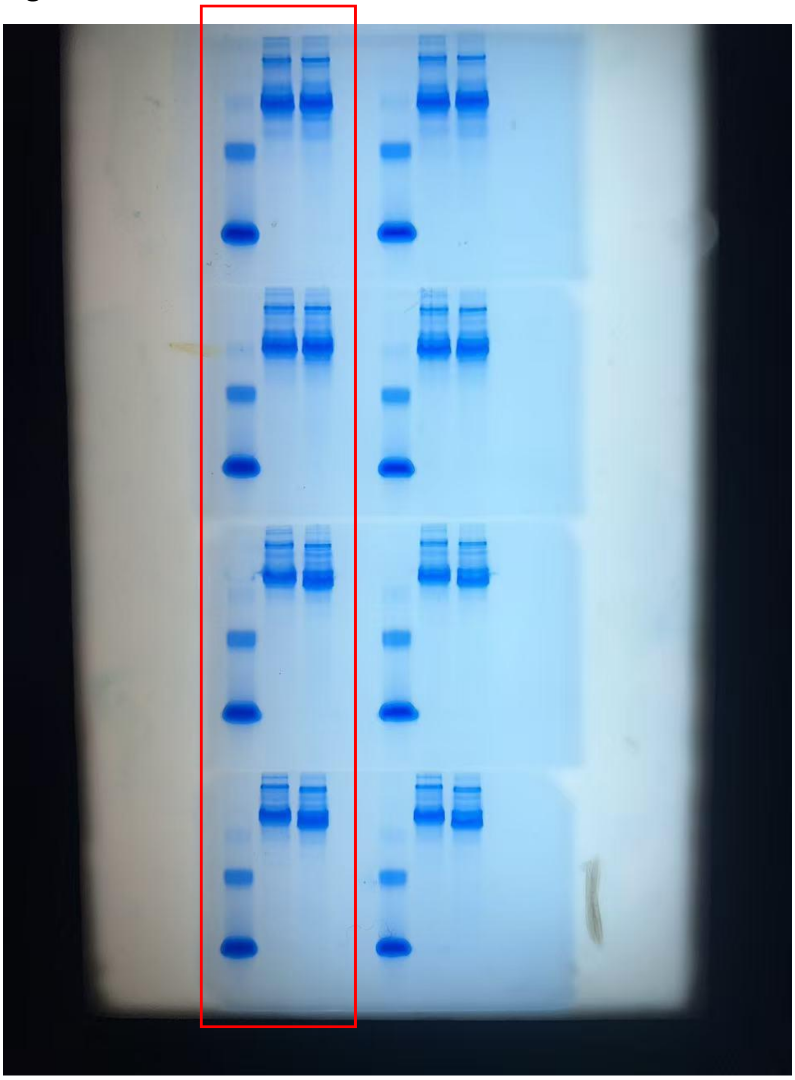

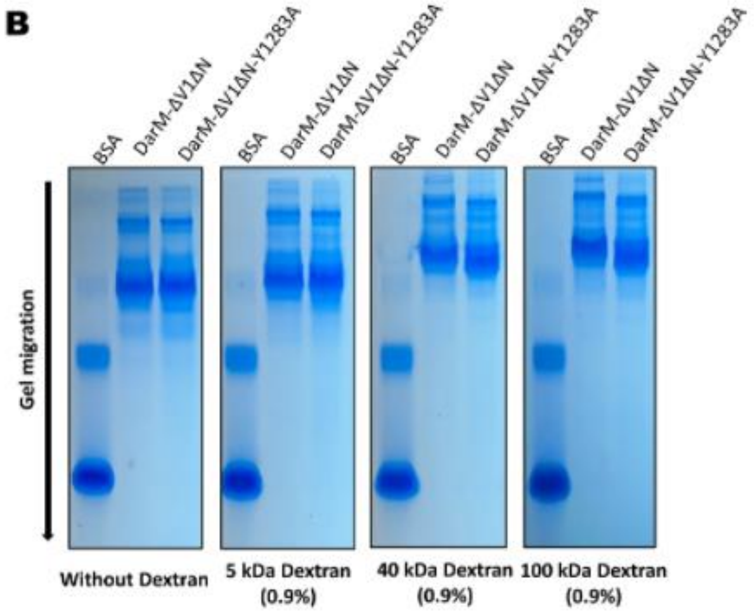
B
BSA
DarM-ΔV1ΔN
DarM-ΔV1ΔN-Y1283A
BSA
DarM-ΔV1ΔN
DarM-ΔV1ΔN-Y1283A
BSA
DarM-ΔV1ΔN
DarM-ΔV1ΔN-Y1283A
BSA
DarM-ΔV1ΔN
DarM-ΔV1ΔN-Y1283A
Gel migration
Without Dextran
5 kDa Dextran (0.9%)
40 kDa Dextran (0.9%)
100 kDa Dextran (0.9%)
