# Supplementary figures and images for: One-step enzymatic synthesis of medium molecular weight dextran using engineered dextransucrase DarM from Leuconostoc citreum CBA3623
Source: Front Microbiol. 2026 Jun 3;17:1833544. doi: 10.3389/fmicb.2026.1833544 (PMC13272390; doi:10.3389/fmicb.2026.1833544)

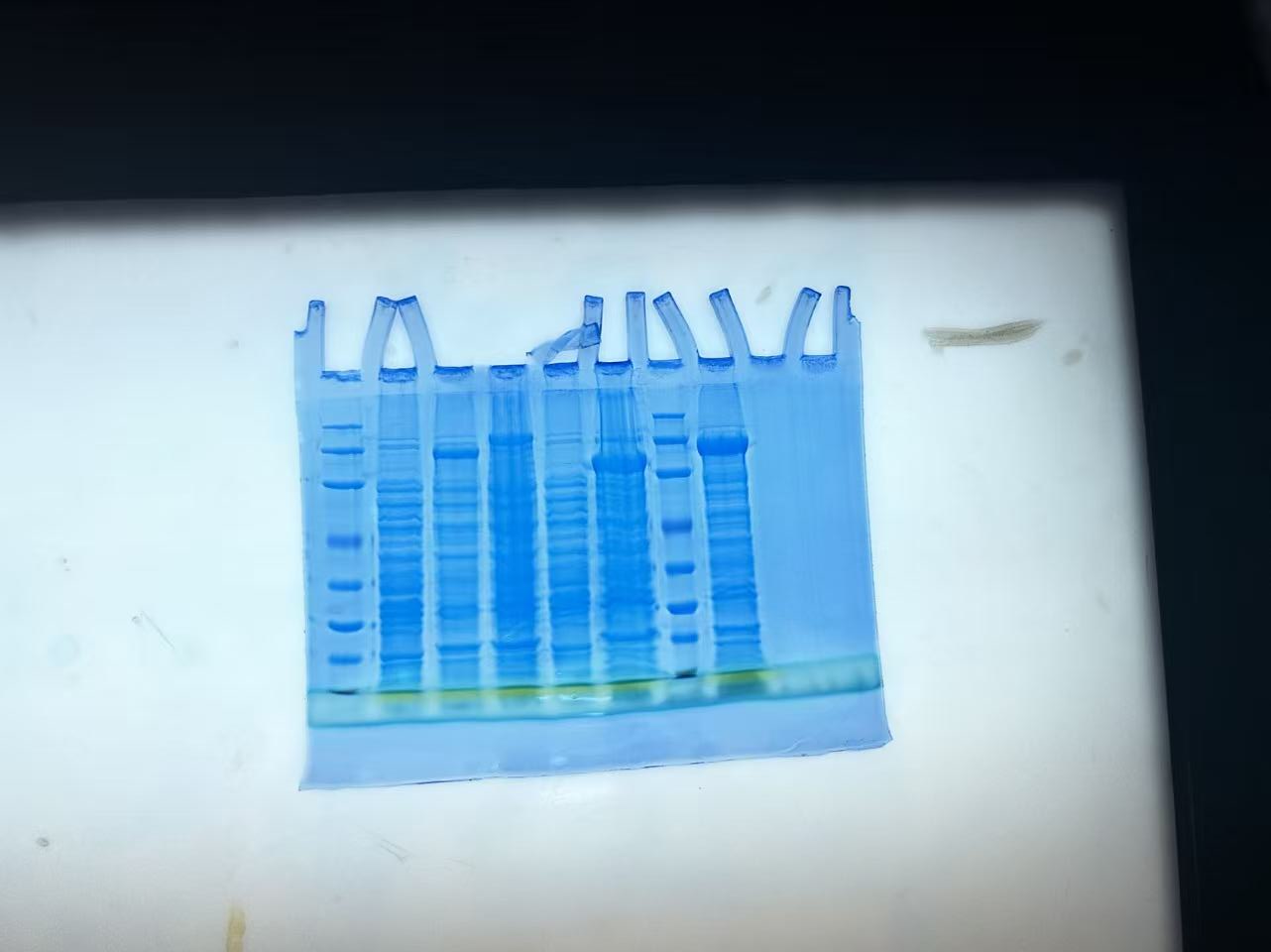

Supplement: Supplementary file 1 [file Data_Sheet_1.zip › Proof-Original Gel images/Original Figure 2B Left.tif]

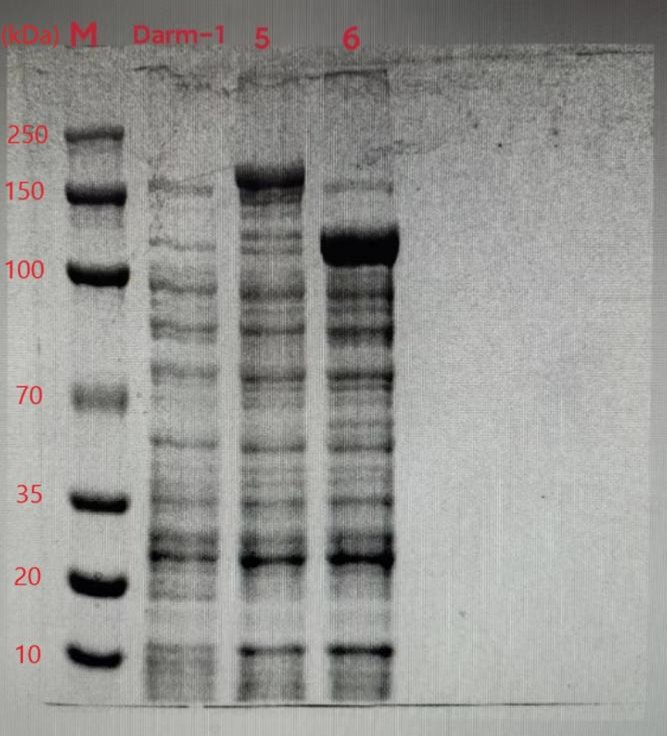

Supplement: Supplementary file 1 [file Data_Sheet_1.zip › Proof-Original Gel images/Original Figure 2B Right.tif]

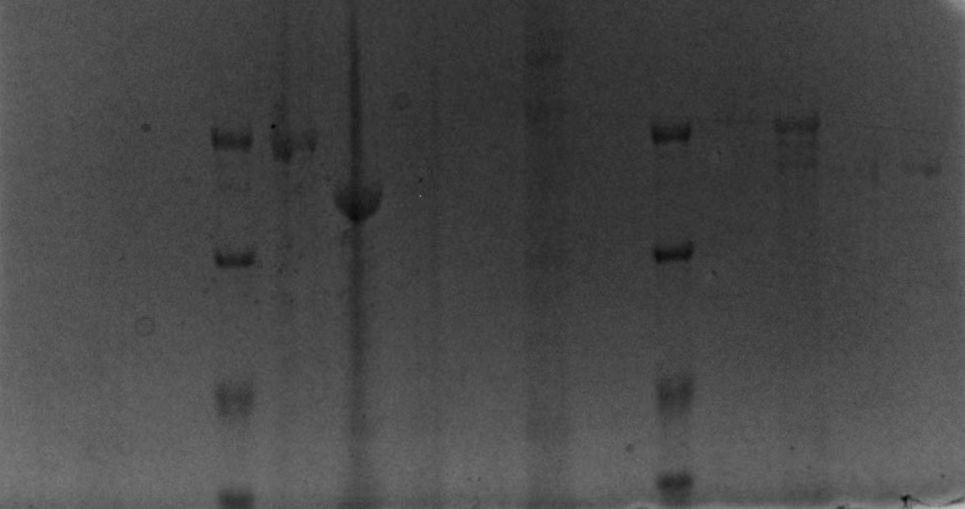

Supplement: Supplementary file 1 [file Data_Sheet_1.zip › Proof-Original Gel images/Original Figure 2C.tif]

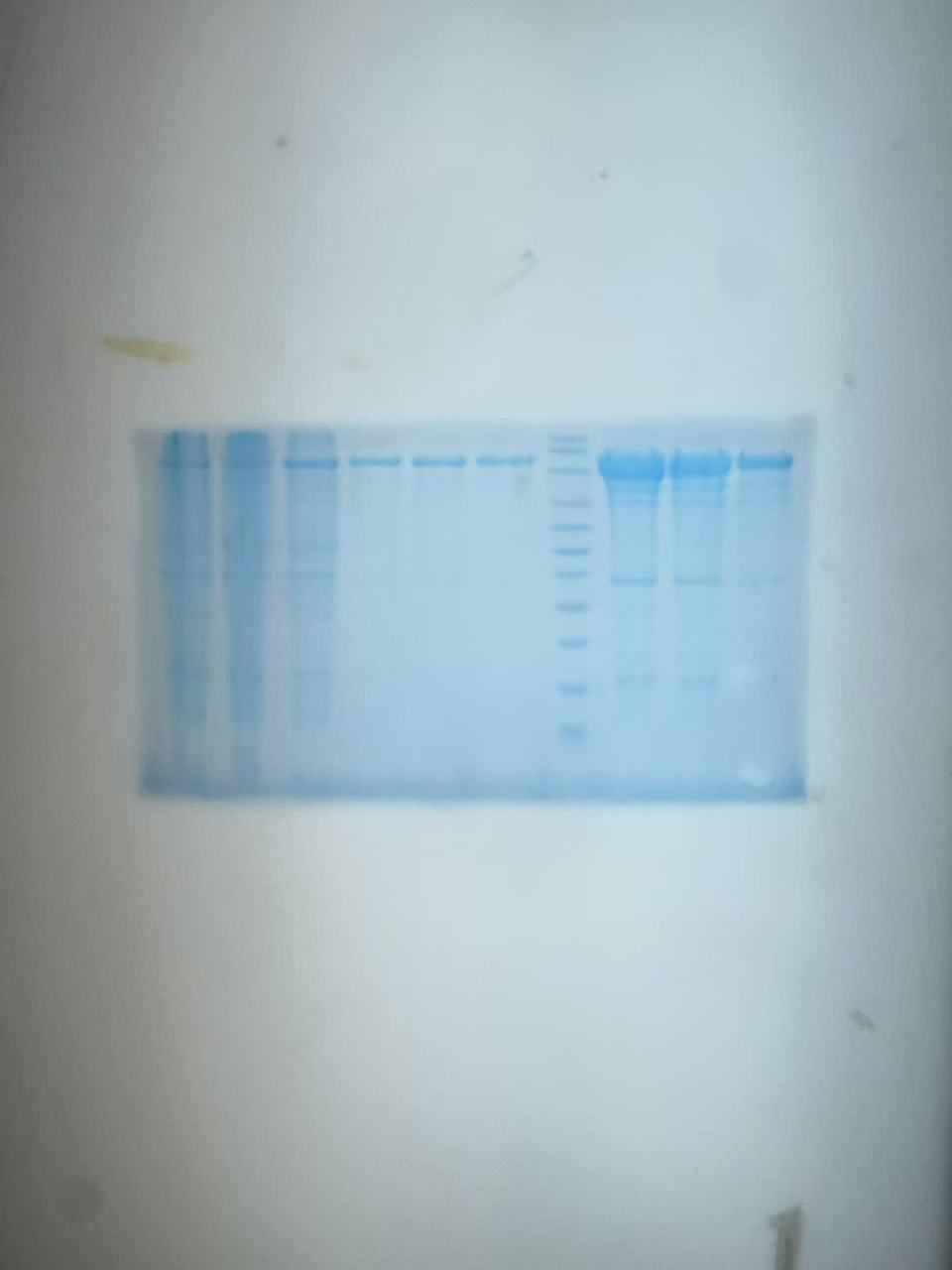

Supplement: Supplementary file 1 [file Data_Sheet_1.zip › Proof-Original Gel images/Original Figure 6A.tif]

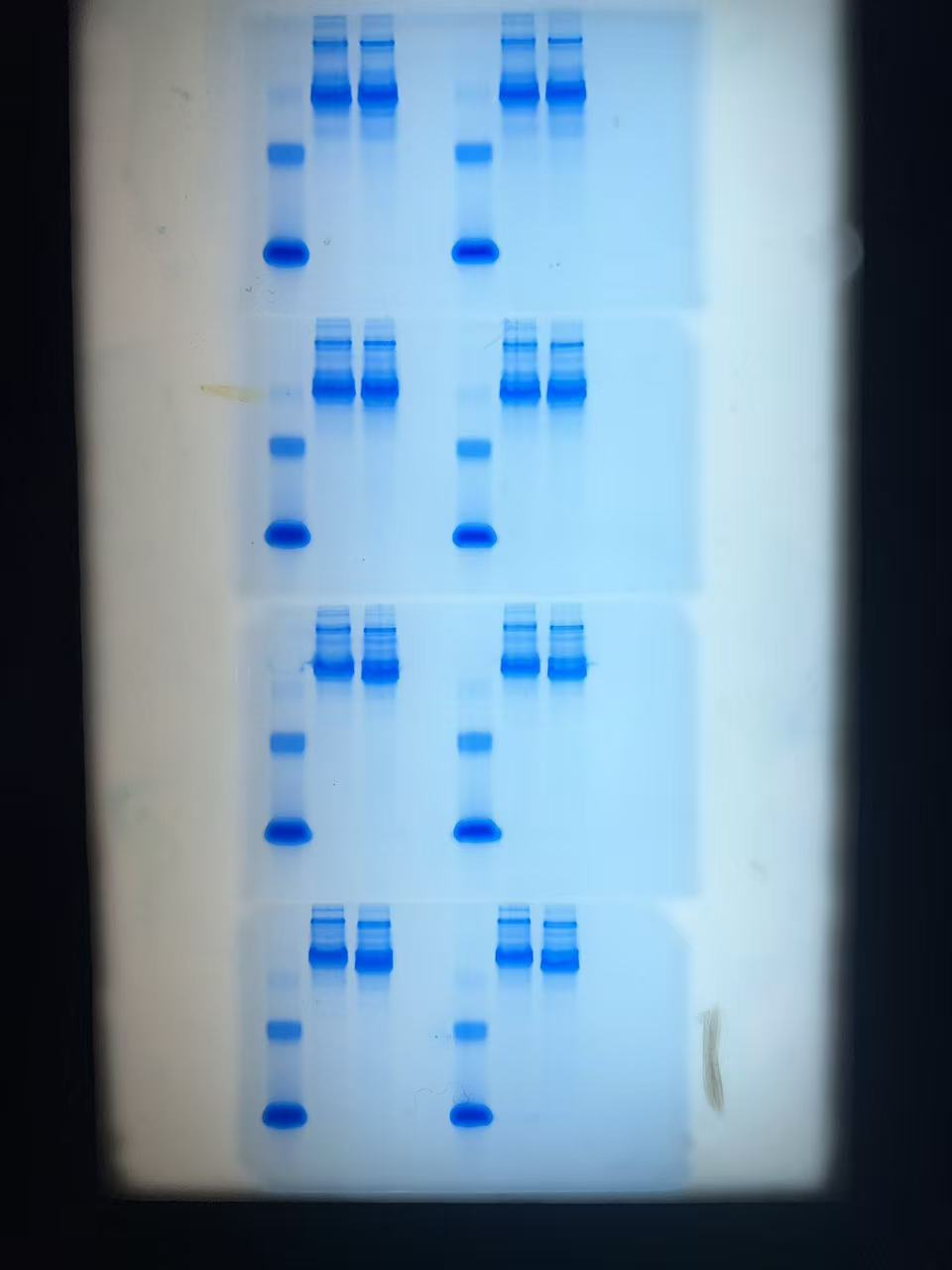

Supplement: Supplementary file 1 [file Data_Sheet_1.zip › Proof-Original Gel images/Original Figure 6B.tif]
