# Supplementary material for: One-step enzymatic synthesis of medium molecular weight dextran using engineered dextransucrase DarM from Leuconostoc citreum CBA3623
Source: Front Microbiol. 2026 Jun 3;17:1833544. doi: 10.3389/fmicb.2026.1833544 (PMC13272390; doi:10.3389/fmicb.2026.1833544)
Supplement: Supplementary file 2 [file Table_1.docx]

**Table S1.** Representative GSs from different genus sources.

| **GS** | **Strain** | **Name** | **GenBank** |
| --- | --- | --- | --- |
| Dextransucrase | *Leuconostoc citreum* CBA3623 | DarM | This work |
|  | *[Leuconostoc mesenteroides](https://www.ncbi.nlm.nih.gov/protein/AAD10952.1)* NRRL B 512F | DSR-S | AAD10952.1 |
|  | [*Leuconostoc mesenteroides*](https://www.ncbi.nlm.nih.gov/protein/AAG61158.1) Lcc4 | DSR-D | AAG61158.1 |
|  | *Leuconostoc mesenteroides* 0326 | DEX-YG | ABC75033.1 |
|  | *Leuconostoc mesenteroides* B 1299CB4 | DSR-BCB4 | ABF85832.1 |
|  | *Leuconostoc mesenteroides* IBUN 91.2.98 | DSR-IBUN | WKU61625.1 |
|  | *Leuconostoc mesenteroides* strain IBT-PQ | DSR-P | AAS79426.1 |
|  | *Leuconostoc mesenteroides* L0309 | DSR-X | AAQ98615.2 |
|  | *Leuconostoc*  *mesenteroides NN710* | LmDexA | OP778186.2 |
|  | *Leuconostoc citreum* NRRL B-1299 | DSR-A | CDX67012.1 |
|  |  | DSR-B | CDX65712.1 |
|  |  | DSR-M | CDX66895.1 |
|  |  | DSR-DP | CDX66641.1 |
|  |  | DSR-E | CDX66820.1 |
|  | *Leuconostoc citreum* B/110-1-2 | DSR-F | ACY92456.2 |
|  | *Lactobacillus reuteri*  180 | GTF-180 | AAU08001.1 |
|  | *Lactobacillus sakei* Kg15 | GtfKg15 | AAU08011.1 |
|  | *Lactobacillus fermentum* Kg3 | GtfKg3 | AAU08008.1 |
|  | *Lactobacillus parabuchneri* 33 | Gtf33 | AAU08006.1 |
|  | *Weissella cibaria* CMU | DSRWC | ACK38203.1 |
|  | *Streptococcus oralis* ATCC 10557 | GTFR | BAA95201.1 |
| Alternansucrase | *Leuconostoc mesenteroides* NRRL B 1355 | ASR | CAB65910.2 |
|  | *Leuconostoc citreum* ABK-1 | LcALT | AIM52834 |
| Mutansucrase | *Streptococcus mutans* GS5 | GtfD (GTF-S) | WP_002289671.1 |
|  | *Streptococcus mutans* GS5 | GTFB (GTF-I), | AAA88588.1 |
|  | *Streptococcus mutans* GS5 | GTFC (GTF-SI) | AAA88589.1 |
|  | *lactobacillus reuteri* ML1 | GTFML1 | AAU08004.1 |
| Reuteransucrase | *lactobacillus reuteri* 121 | GTFA | AAU08015.1 |
|  | *lactobacillus reuteri*  ATCC 55730 | GTFO | AAY86923.1 |

**Table S2.** Primers of dextransucrase variants.

| Primer name | Primer 5’-3’ | Amplification region (amino acid position) |
| --- | --- | --- |
| *darM-***ΔAPY**-F | atgggtcgcggatccgaattcATGAAAATAAAAGAAACAATTACCCG | aa1-aa1370 |
| *darM-***ΔAPY**-R | ctcgagtgcggccgcaagcttCACACCTAAAGTTGACGAAGTATCAA |  |
| *darM-***ΔSP**-F | atgggtcgcggatccgaattcATGATATCGAATGTAGATGGTGTTATAAAC | aa45-aa1935 |
| *darM-***ΔSP**-R | ctcgagtgcggccgcaagcttTTAAGCTTGCAAAGCACGCTT |  |
| *darM-***ΔV1**-F (**ΔV1ΔC**-F) | gtgccgcgcggcagccatatgATGACAACACAGAGCCAACAGGA | aa45-aa1370 |
| *darM-***ΔV1**-R (**ΔV1ΔN**-R) | gtggtggtggtggtgctcgagTTACACACCTAAAGTTGACGAAGTATCA |  |
| *darM-***ΔV2**-F (**ΔV1ΔN**-F) | atgggtcgcggatccgaattcATGGGCGGTCGATACGAAC | aa171-aa1302 |
| *darM-***ΔV2**-R (**ΔV1ΔC**-R) | ctcgagtgcggccgcaagcttGGTAGACATTGTTCCATCAGAATTCA |  |
| *darM-***Y1283A** -F | atgttaactaatcaaGCtgtatttgatgcaagcggg | aa171-aa1370 |
| *darM-***Y1283A**-R | cccgcttgcatcaaatacaGCttgattagttaacat |  |


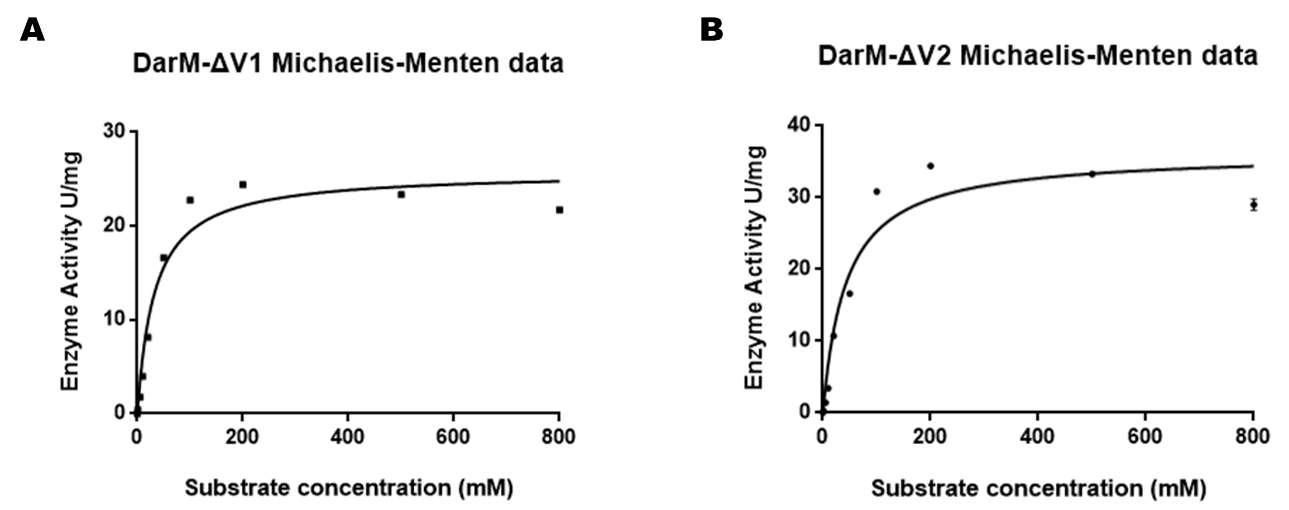

**Figure S1. Michaelis-Menten kinetic analysis of DarM-ΔV1 and DarM-ΔV2.** Initial velocities were measured using the DNS assay at sucrose concentrations of 0-800 mM under the respective optimal pH and temperature conditions. Data are shown as mean ± SD (n = 3), and the solid lines represent nonlinear fits to the Michaelis-Menten equation.


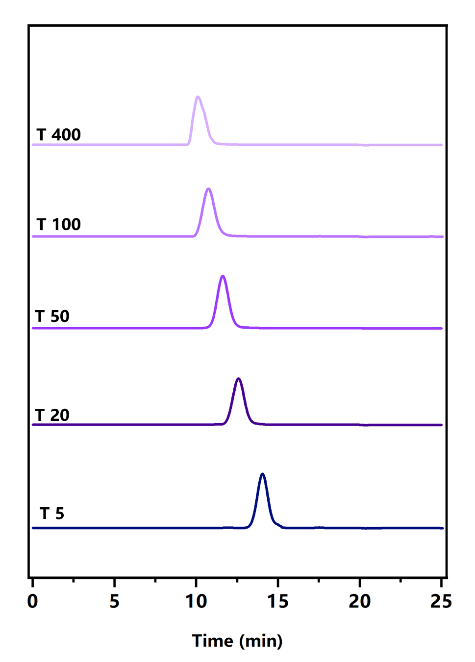


**Figure S2.** HPLC chromatograms of dextran standard.


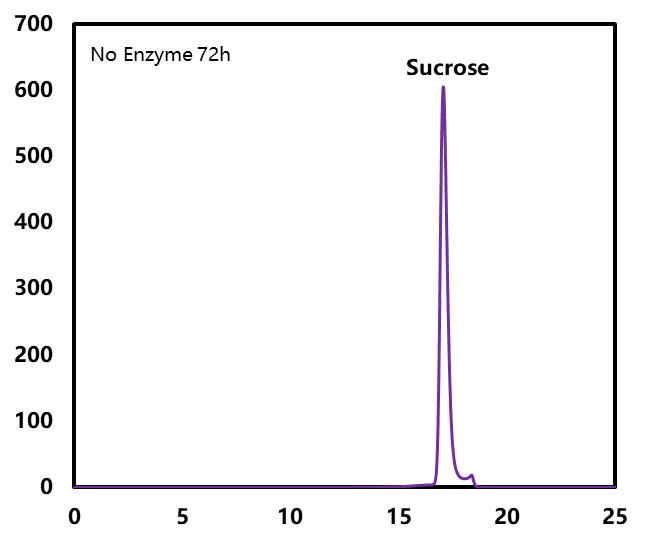


**Figure S3.** HPLC chromatogram of the no-enzyme control.


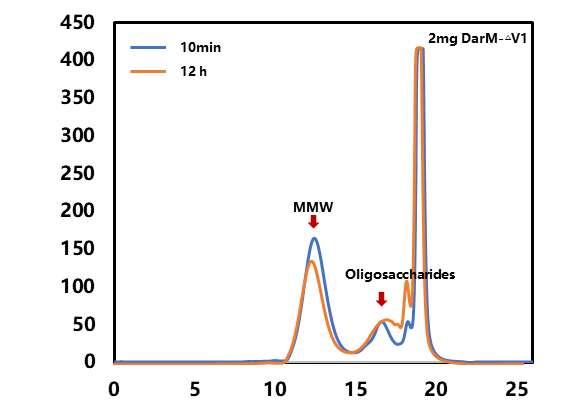


**Figure S4.** Time-course HPLC chromatograms of products synthesized by DarM-ΔV1 at 2mg/mL final enzyme concentration.


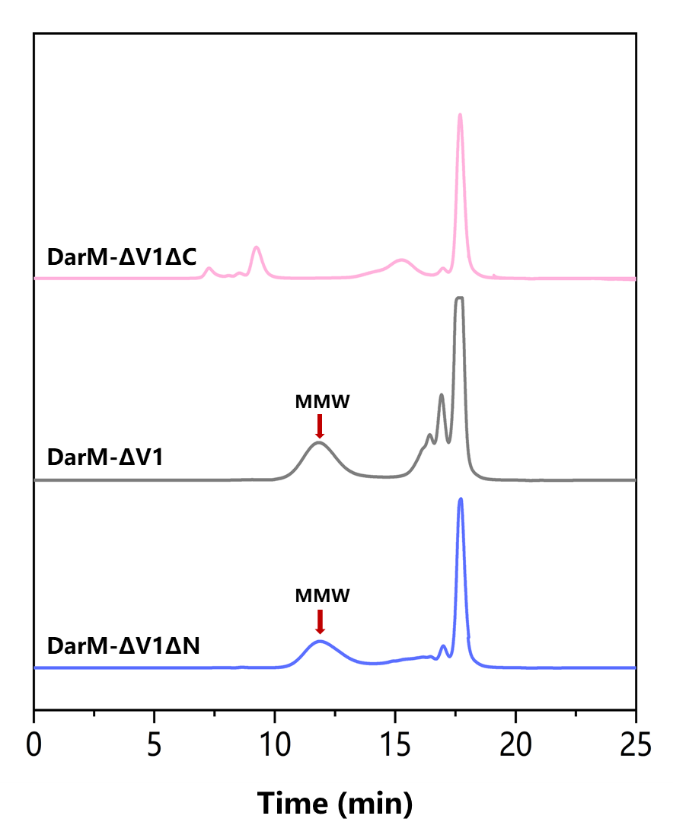


**Figure S5.** HPLC chromatograms of products synthesized by DarM-ΔV1, DarM-ΔV1ΔN (residues 171–1370), and DarM-ΔV1ΔC (residues 45–1302)


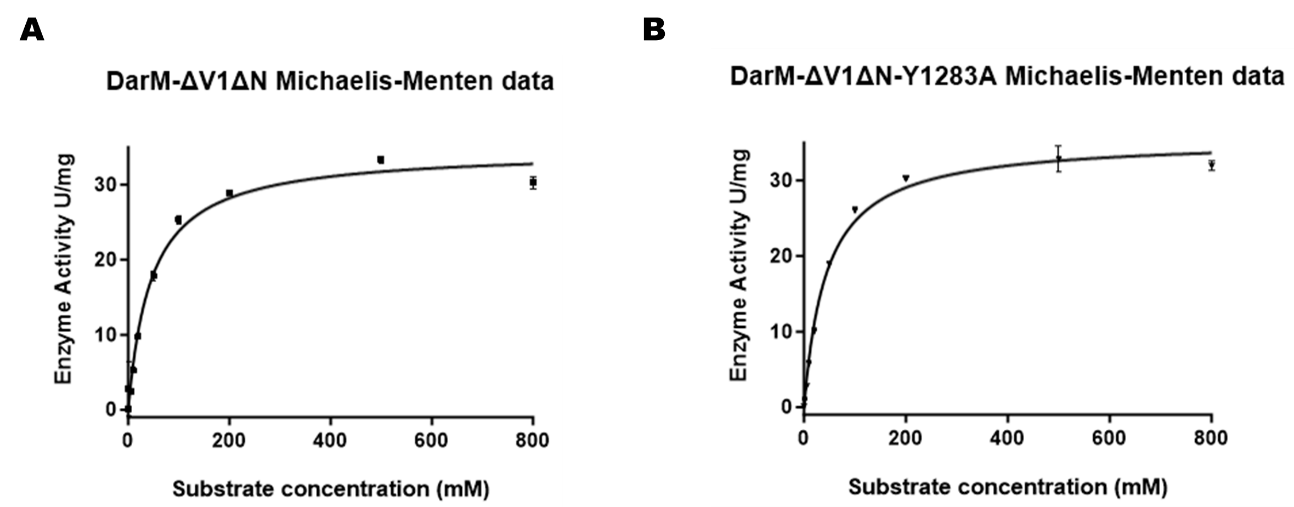


**Figure S6. Michaelis-Menten kinetic analysis of DarM-ΔV1ΔN and DarM-ΔV1ΔN-Y1283A.** Initial velocities were measured using the DNS assay at 25°C over a sucrose concentration range of 0-800 mM. Data are shown as mean ± SD (n=3), and the solid lines represent nonlinear fits to the Michaelis-Menten equation.
